# Characterization of post-COVID syndrome by self-perceived symptom severity stratified by infection wave: *beyond COVID,* a prospective, multicenter cohort study in Germany

**DOI:** 10.1007/s15010-026-02819-6

**Published:** 2026-05-18

**Authors:** Alexander Mertens, Judith Smith, Ingmar Bergs, Julia Fischer, Sarah Jansen, Sven Breitschwerdt, Elisabeth Pracht, Alexander Killer, Lukas Schipper, Nils Kuklik, Mirjam Frank, Julia Schwichtenberg, Siona Bührmann, Lina Zeissler, Michael Dreher, Jürgen Rockstroh, Hana Rohn, Clara Lehmann, Phil-Robin Tepasse, Börge Schmidt, Nico Dragano, Johannes Bode, Tom Luedde, Björn-Erik-Ole Jensen

**Affiliations:** 1 Department of Gastroenterology, Hepatology and Infectious Diseases, Medical Faculty of Heinrich Heine, University Hospital Düsseldorf, University Düsseldorf, Moorenstraße 5, 40225 Düsseldorf, Germany; 2 Department of Pneumology and Intensive Care Medicine, RWTH Aachen University Hospital, Aachen, Germany; 3 Department of Internal Medicine B, Hepatology, Endocrinology and Clinical Infectiology, University Hospital Münster, Münster, Germany; 4 Department of Infectious Diseases, West German Centre of Infectious Diseases, University Hospital Essen, University of Duisburg-Essen, Essen, Germany; 5 Department of Medicine I, Bonn University Hospital, Bonn, Germany; 6 Department I of Internal Medicine, Faculty of Medicine and University Hospital Cologne, University of Cologne, Cologne, Germany; 7 Institute for Medical Informatics, Biometry and Epidemiology, University Hospital Essen, University Duisburg-Essen, Essen, Germany; 8 Medical Faculty and University Hospital, Institute of Medical Sociology, Centre for Health and Society, Heinrich Heine University Düsseldorf, Düsseldorf, Germany; 9 Centre for Clinical Trials Essen, University Hospital Essen, Essen, Germany

**Keywords:** PCS, Long COVID, Long COVID-19, Post COVID, Post COVID Condition, PCC, COVID-19, Follow-up after COVID-19, COVID-long-term, Gender association in post-COVID-19, Variant of Concern, VOC, VOCs, Wave-association, Persistence of COVID-19 symptoms, SARS-CoV-2, Post acute sequelae of SARS CoV-2 infection, COVID-waves, Alpha, Delta, Omicron

## Abstract

**Background:**

Post-COVID syndrome (PCS) refers to persistent or new-onset symptoms 3 months after SARS-CoV-2 infection lasting for at least 2 months. The characterization of PCS varies across studies, with a substantial heterogeneity regarding different study samples, survey instruments, and follow-up periods. This is particularly the case in hospitalized patients vs. outpatients, as well as regarding different variants of concern (VOCs) and their impact on the frequency and severity of specific symptoms.

**Methods:**

The study population of *Beyond COVID* was recruited in six German cities by inviting (1) individuals registered as SARS-CoV-2 PCR positive at the local Public Health Authorities and (2) previously hospitalized patients with infection date after 1st March 2021. Participants were allocated to the predominant VOC of their first infection. Questionnaires to assess pre-existing conditions, symptoms during infection, and persisting symptomswere performed. Follow-up at sixth-month intervals is planned for 3 years. The study is ongoing. This publication describes the parameters at the baseline visit (BV).

**Results:**

We included 1257 participants (13% hospitalized-based; 87% population-based). Most participants had BA.2 (n = 436; 35%), followed by Delta (n = 336; 27%), BA.1 (n = 238; 19%), and Alpha (n = 218; 17%). The mean age was 47.1 years, and 59% of the participants were female. 72% reported at least one persisting symptom. Fatigue was the most frequent ongoing symptom (33%), followed by concentration disorders (25%) and dyspnoea (22%). Female sex, lower education, and a shorter period between infection and BV were associated with higher rates of persisting symptoms and symptom severity. Among all VOCs, participants with BA.1 and BA.2 had the lowest rates of persisting symptoms and symptom severity.

**Conclusion:**

Analysis of baseline data from the Beyond-COVID cohort confirms a high percentage of persistent symptoms. Omicron variants had lower rates of persistent symptoms and symptom severity. We are confident, that long-term follow-up and the additional results from our clinical examinations, blood samples, sociodemographic and psychosocial data will further contribute to the characterization of PCS.

## Introduction

By December 2024, the COVID-19 pandemic led to over 777 million confirmed cases [1]. Early on in the SARS-CoV-2 pandemic, patients with persistent symptoms after recovery of COVID-19 were described. The WHO specifies that symptoms after COVID-19 “may be new onset, following initial recovery from an acute COVID-19 episode, or persist from the initial illness. Symptoms may also fluctuate or relapse over time” [2]. The WHO (World Health Organization) definition uses the term *post-COVID-19 condition (PCC)* for symptoms lasting at least 8 weeks, whereas NICE (National Institute for Health and Care Excellence, UK) defines *post-COVID-19 syndrome* as symptoms lasting at least 12 weeks [2, 3]. Both definitions include the requirement that symptoms must be present at least 12 weeks after the SARS-CoV-2 infection, and both definitions separate from the term long COVID, which is defined as signs and symptoms of COVID-19 from 4 weeks up to 12 weeks [3]. Furthermore, the terms p*ost-COVID syndrome (PCS)* and *post COVID* are used as umbrella terms for both post-COVID-19 syndrome and *post-COVID-19* condition, as most studies did not apply the definitions consistently [4, 5]. For readability and due to the interchangeability of the terms PCS and post COVID [5], we use the term post COVID in the following. The WHO and the NICE definitions are inclusive and acknowledge that up to 200 different post COVID symptoms have been described, and that confirmation of SARS-CoV-2 infection is not mandatory since it is not feasible in some regions (e.g., countries with limited access to healthcare) [6].

The reported prevalence of post COVID widely differs across studies and can be estimated at around 10–30% in most studies, with an extensive range of 2–80% [7–10]. The Global Burden of Disease Long COVID study included 1.2 million people who had experienced an acute symptomatic SARS-CoV-2 infection and reported up to 15.1% long-lasting symptoms one year after infection [11]. Variation in post COVID prevalence and symptom-complex definition in the literature is to a large extent due to a lack of a uniform applied definition, the differences in observational time by studies as well as the consideration of various variants of concern (VOCs), and the characteristics of participants included in each study (asymptomatic vs. symptomatic non-hospitalized vs. hospitalized participants vs. a mixture) [10, 12]. In addition, many studies refer to data from highly pre-selected samples of patients referred to the participant centers or limited to online surveys [13, 14], which are potentially biased (e.g., by self-referrals).

Regarding SARS-CoV-2 variants, the virus emerged from the original wild-type strain to Alpha (B.1.1.7), Beta (B.1.351), Delta (B.1.617.2), and Omicron (B.1.1.529) over time, with defined time periods for each dominant variant [14, 15]. The impact of different SARS-CoV-2 variants remains debatable, with most studies limited to retrospective data and online surveys [16, 17].Furthermore, the majority of published studies to date focus on the characterization of one or two virus variants and a specific, circumscribed topic (e.g. infectivity/transmission, hospital admission rate, influence of vaccination), as shown in the synthesis of results in the systematic reviews by Vishwakarma et al. and Du et al., stating that the difference in the prevalence of symptoms and post COVID between various variants still needs to be explored [14, 15, 18–24].The present study aims to provide further evidence regarding post COVID symptoms and symptom severity following infection from different periods throughout the pandemic phases. It adds relevant data to other comparable approaches [25]: By regarding a systematic survey of post COVID and global symptom severity by examining randomly selected COVID-19 infected individuals from the general population together with a hospital-based sample regardless of the symptoms during or after the infection and stratified by the predominant virus variants.

## Materials and methods

### Study population

This prospective multicenter cohort study includes SARS-CoV-2-positive tested (PCR) individuals who met the inclusion criteria (see below). Six study centers (University Hospitals of Aachen, Bonn, Essen, Düsseldorf, Cologne, and Münster), all located in the federal state of North Rhine-Westphalia, took part. Two samples were defined: Individuals with laboratory-confirmed COVID-19 infection registered at the local Public Health Authorities (population sample) and hospitalized patients, registered at the participationg study centers (hospital based). The public health authorities drew consecutive samples from the registration data until the planned number, as specified in the study protocol, was reached, and limited the samples to infections that occurred after the cut-off date of 1 March 2021.

The second sample consisted of all patients hospitalized in one of the six participating clinics after the same cut-off date for the infection (1 March 2021). In this case, contact was made directly by the participating study centers. Recruitment (in both samples) was conducted between 01/2022 and 09/2023.

Inclusion Criteria

- Main residence in North Rhine-Westphalia in the catchment area of the cities of Aachen, Bonn, Düsseldorf, Essen, Cologne or Münster.
- 18–75 years old.
- Evidence of a survived SARS-CoV-2 infection (PCR test).
- Infection after March 1st 2021.
- The time of one infection was at least 3 months ago (reference date: date of the first positive PCR test).
- Patients with active COVID-19 infection or an infection that occurred less than 3 months ago were scheduled for later inclusion in the study.
- The person was able to take part in the survey and the examination, and has sufficient knowledge of the German language or support from an interpreter.

### Study baseline and follow-up

**Fig. 1:**
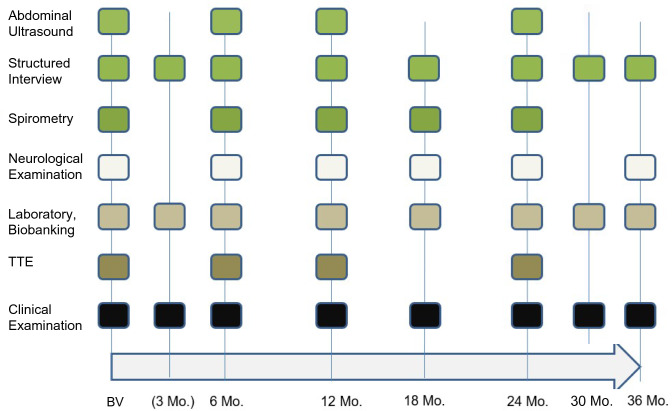
Study protocol: Overview of the type of examinations carried out and their time interval. *BV* Baseline Visit, *TTE* transthoracic echocardiography

Both cohorts (population- and hospital-based) were examined at the Post-COVID-19 clinic of each center. Blood, urine, and saliva samples were taken following participant information and informed consent. Furthermore, participants underwent medical examinations by a study physician. Neurological tests, spirometry, transthoracic echocardiography, and abdomen ultrasound were performed (Fig. 1). Aroma and taste tests were performed at the initial visit and, if pathological, on the following appointments according to the study protocol. Participants responded to questionnaires to assess general health-related information, persisting (severity and duration) symptoms, quality of life, disability, and sociodemographic and psychosocial factors at each visit. Questionnaires collected data on the date of the first and subsequent infections, vaccination, and new health issues. Follow-up visits were conducted at 3 (optioal), 6,12, 18, 24, 30, and 36 months after the BV, Fig. 1. The Follow-up is is ongoing; the results of these examinations will be published upon completion.

### Data assessment

#### Pre-existing conditions

Pre-existing conditions, defined as conditions that existed before SARS-CoV-2 infection in participants’ medical histories, were ascertained from information provided by participants via questionnaires, verbally, or in medical documentation and were subsumed into categories: Overweight/Adipositas; Cardiovascular Disease; Thyroid Dysfunction; Neurological Disorder; Psychiatric Disorder; Muscoloskeletal Disease; Chronic Respiratory Disease; Hypercholesterolemia; Autoimmune Disease; Diabetes mellitus; Malignant Tumor; Steatosis hepatis; Upper Gastrointestinal Disease; Sleep Apnea; Infectious Disease; Immunsuppression; Chronic Kidney Disease; Pancreatico Biliary Disease; Irritable Bowel Syndrome; Chronic Inflammatory Bowel Disease; Liver Cirrhosis.

#### Persistent symptoms

Persistent symptoms were assessed by questionnaire. Persistent symptoms were defined as reporting at least one symptom due to the participants’ first SARS-CoV-2 infection still present at the baseline examination. Participants were asked for persistent symptoms that occurred initially during the acute infection and persistent symptoms that occurred later.

#### Persistent symptom severity level

The overall severity level of persistent symptoms was assessed on a scale from 0 to 10, and the participants were asked how severely they were affected by the reported symptoms at the time of the baseline examination. The severity level of participants who reported no persistent symptoms was coded 0.

#### Frequency of persisting symptoms regarding VOC and symptom-cluster

Questionnaires assessed 25 different symptoms: fatigue, headache, concentration disorders, psychiatric disorders, dyspnoea, coughing, chest pain, sore throat, voice change, dysgeusia, dysosmia, dizziness, tinnitus, earache, nausea, loss of appetite, diarrhea, constipation, abdominal pain, limb pain, back pain, fever, chill, cold, and hair loss.

We subsumed the frequencies of reported ongoing single symptoms into five predefined clusters [26, 27]: chronic fatigue-like (fatigue, headache, concentration disorders), respiratory (dyspnoea, coughing, chest pain, sore throat, voice change), neurosensorial (dysgeusia, dysosmia, dizziness, tinnitus, earache), gastrointestinal (nausea, loss of appetite, diarrhoea, constipation, abdominal pain), and systemic-inflammatory chronic pain (limb pain, back pain, fever, chill, cold, hair loss). Psychiatric disorders were excluded from cluster allocation. As allocating symptoms based on previously conducted cluster analyses was not possible (this was the case for the symptoms hair loss and cold), allocation was based on published data [28–31].

#### Time from infection to study baseline

The time from the first infection to the study baseline was calculated for each participant and included as a continuous variable (days) to account for differences in time for recovering from SARS-CoV-2 infection.

#### Vaccination before the first infection

The vaccination history of the participants was recorded in a face-to-face interview, and we determined if a vaccination occurred before or after the first infection.

#### SARS-CoV-2 wave of infection (VOCs)

The date of the first infection was used to assign participants to certain VOCs according to the different periods of COVID-19 in Germany [15]. This resulted in a categorical variable with five categories. The first category consisted of participants with an infection between the 9th and the 30th calendar week of 2021, representing the third COVID wave in Germany (VOC: Alpha). The second category comprises the fourth infection wave, i.e., the period from the 31st–51st calendar week of 2021 (VOC: Delta). The third, fourth and fifth category representing sublines of Omicron: the third extends from the 52nd calendar week of 2021 to the 8th calendar week of 2022 (VOC: Omicron BA.1). Category 4 extends from the 9th calendar week of 2022 to the 21st calendar week of 2022 (VOC: Omicron BA.2). Category 5 covers the period from the 22nd calendar week of 2022 (VOC: Omicron BA.5).

#### Educational attainment

Educational attainment was assessed as the highest general school-leaving qualification (high school graduation) and dichotomized into a variable indicating whether a general or subject-specific tertiary education entrance qualification was attained.

#### Multiple infections

Participants provided information on every confirmed SARS-CoV-2 infection. Information was dichotomized into one confirmed infection vs. more than one confirmed infection.

### Statistical analysis

Descriptive statistics were calculated as contingency tables. In the descriptive statistics, unadjusted frequencies are reported, which could be subject to confounding by age, sex, and other covariates. Participants with additional missing values in the covariates were only excluded from the analyses that included covariates. Logistic regression models (with n = 1117 complete observations) were fitted to calculate odds ratios (ORs) and 95%-confidence intervals (95%-CIs) for the association of VOCs with persistent symptoms due to the first SARS-CoV-2 infection. Models were adjusted for the potential confounders: vaccination before the first infection, study center, educational attainment, multiple infections, and time from the first infection to the study baseline. In addition, linear regression models were fitted with the same covariates to calculate beta estimates and 95%-CIs for the association of SARS-CoV-2 infection waves with severity level due to the first SARS-CoV-2 infection at baseline examination. The number of observations also amounted to n = 1117. We have included study center as a fixed effect in the model, as with only six centers fitting a generalized linear mixed model with a random intercept for study center may have led to poor estimation of between-cluster variance and potential model non-convergence. Given the primarily exploratory observational character of the study, we did not set an alpha level for dichotomizing p-values in the regression models. To assess differences in the frequency of symptoms regarding different VOC, the chi-squared test and Fisher’s exact test were applied. Statistical tests were two-sided and a p-value < 0.05 was considered significant. Analyses were conducted using SAS version 9.4 (SAS Institute) and the spreadsheet program Excel (Microsoft Corporation, Redmond, WA, USA).

## Results

### Study population

A total of 1185 individuals participated from the first sample (response rate: 4.7%). In the second sample, 199 participants took part (response rate: 11.4%). Ten participants (0.7%) were excluded (missing informed consent; not meeting the inclusion criteria). Overall, 1373 participants were included in the analysis. Of those, 1257 had complete information for statistical analysis on infection wave stratification, persistent symptoms (related to the first infection), and the corresponding severity level, of which 87% (n = 1088) were from the population-based sample. Table 1 presents the main characteristics of the sample stratified by infection wave. The median time from first infection to BV was 382 days. Most participants were female (n = 740; 59%), and the mean age was 47.1 (± 14.5) years. Regarding education level, 59% (n = 725) had at least a high school graduation.

**Table 1:** Study population stratified by infection Wave/VOC

Variable	1st–3rd Wave VOC: Alpha	4th Wave VOC: Delta	5.1th Wave VOC: BA.1	5.2th Wave VOC: BA.2	6th Wave VOC: BA.5	total sample
N	218 (100%)	336 (100%)	238 (100%)	436 (100%)	29 (100%)	1257 (100%)
Age (years; mean)	47.4 (± 13.2)	47.4 (± 14.3)	45.9 (± 15.5)	47.4 (± 14.8)	46.1 (± 12.0)	47.1 (± 14.5)
Sex male	99 (45%)	131 (39%)	96 (40%)	181 (42%)	10 (34%)	517 (41%)
Sex female	119 (55%)	205 (61%)	142 (60%)	255 (58%)	19 (66%)	740 (59%)
High School Graduation	108 (51%)	205 (62%)	130 (57%)	262 (61%)	20 (71%)	725 (59%)
Hospital-based	45 (21%)	49 (15%)	34 (14%)	38 (9%)	3 (10%)	169 (13%)
Population-based	173 (79%)	287 (85%)	204 (86%)	398 (91%)	26 (90%)	1088 (87%)
Number of Participants with ≥ 1 Pre-Existing Condition	182 (83%)	284 (85%)	194 (82%)	347 (80%)	24 (83%)	1031 (82%)
Number of Pre-Existing Conditions (mean)	2.8 (± 1.8)	2.6 (± 1.9)	2.7 (± 1.7)	2.6 (± 1.8)	2.6 (± 1.6)	2.6 (± 1.8)
Number of Vaccinated Participants (before 1st Infection)	30 (15%)	284 (96%)	213 (99%)	417 (100%)	26 (100%)	970 (84%)
*Number of Infections at BV*						
One	130 (60%)	217 (65%)	157 (66%)	329 (75%)	26 (90%)	859 (68%)
Two	83 (38%)	113 (34%)	79 (33%)	106 (24%)	3 (10%)	384 (31%)
Three	5 (2%)	5 (1%)	2 (1%)	1 (0%)	0 (0%)	13 (1%)
Four	0 (0%)	1 (0%)	0 (0%)	0 (0%)	0 (0%)	1 (0.1%)
Number of Symptoms (during first Infection) (mean)	8.3 (± 5.4)	8.8 (± 5.0)	6.8 (± 4.5)	7.1 (± 4.0)	8.7 (± 4.9)	7.7 (± 4.7)
Number of Participants with ≥ 1 persisting Symptom at BV (first Infection)	111 (51%)	170 (51%)	108 (45%)	186 (43%)	19 (66%)	594 (47%)
Number of persisting Symptoms per Participant (first Infection) (mean)	2.7 (± 2.7)	2.6 (± 3.1)	2.4 (± 3.4)	1.8 (± 2.6)	2.8 (± 3.0)	2.3 (± 3.0)
Severity of Impairment (first infection) (mean)	3.6 (± 2.7)	3.4 (± 2.9)	2.8 (± 2.9)	2.3 (± 2.7)	2.7 (± 2.7)	2.9 (± 2.9)
Number of Participants with ≥ 1 persisting Symptom at BV (all Infections)	190 (87%)	274 (82%)	163 (68%)	256 (59%)	22 (76%)	905 (72%)
Number of persisting Symptoms per Participant (all Infections) (mean)	3.9 (± 3.9)	3.2 (± 3.7)	2.5 (± 3.5)	1.8 (± 2.6)	3.1 (± 3.5)	2.7 (± 3.4)
Severity of Impairment (all Infections) (mean)	3.7 (± 2.7)	3.6 (± 3.0)	2.8 (± 2.9)	2.3 (± 2.7)	2.8 (± 2.7)	2.9 (± 2.9)
Interval 1st Infection – BV (median days, Q1-Q3)	451 (387–537)	367 (304–426)	400 (338–485)	365 (300–413)	226 (146–333)	382 (317–445)
Number of Participants per Study Center						
Center 1	55 (25%)	43 (13%)	65 (27%)	88 (20%)	4 (14%)	255 (20%)
Center 2	18 (8%)	40 (12%)	35 (15%)	94 (22%)	2 (7%)	189 (15%)
Center 3	42 (19%)	70 (21%)	34 (14%)	49 (11%)	6 (21%)	201 (16%)
Center 4	39 (18%)	68 (20%)	41 (17%)	15 (3%)	4 (14%)	167 (13%)
Center 5	45 (21%)	42 (13%)	39 (16%)	53 (12%)	10 (34%)	189 (15%)
Center 6	19 (9%)	73 (22%)	24 (10%)	137 (31%)	3 (10%)	256 (20%)

*BV* baseline visit, *VOC* variant of concern

Most participants had at least one pre-existing condition (82%; n = 1031), with a mean of 2.6 pre-existing conditions, Table 1. The vast majority of participants were vaccinated before the 1st infection (84%; n = 970).

Table 2 presents the main characteristics of the sample stratified by hospital-based (N = 169; 13%) vs. populations-based (n = 1088; 87%). In the population-based sample, the proportion of participants with female sex (60% vs. 50%), with high school graduation (61% vs. 46%), and the proportion of pre-infection vaccinated participants (85% vs. 71%) were higher than in the hospital-based sample. The population sample had a higher average number of symptoms during the first infection compared to the hospital-based sample (7.8 ± 4.7 vs. 7.5 ± 5.2), while the mean severity of impairment was higher in the hospital-based sample (3.6 ± 3.0 vs. 2.8 ± 2.9) regarding first infection and 3.9 ± 3.0 vs. 2.8 ± 2.9 regarding all infections).

**Table 2:** Study population stratified by hospital-based vs. population-based

Variable		Hospital-based Sample (n = 169)			Population-based Sample (n = 1088)	Total (n = 1257)
Age (years; mean)			53.4 (± 14.0)		46.1 (± 14.3)	47.1 (± 14.5)
Sex female Sex male			85 (50%) 84 (50%)		655 (60%) 433 (40%)	740 (59%) 517 (41%)
High School Graduation			73 (46%)		652 (61%)	725 (59%)
Number of Participants with ≥ 1 Pre-Existing Condition			154 (91%)		877 (81%)	1031 (82%)
Number of Pre-Existing Conditions (mean)			3.7 (± 2.4)		2.5 (± 1.6)	2.6 (± 1.8)
Number of Vaccinated Participants (before first Infection) (mean)			100 (71%)		870 (85%)	970 (84%)
Number of Infections at BV						
One			117 (69%)		742 (68%)	859 (68%)
Two			50 (30%)		334 (31%)	384 (31%)
Three			2 (1%)		11 (1%)	13 (1%)
Four			0		1 (0.1%)	1 (0.1%)
Number of Symptoms (during first Infection) (mean)			7.5 (± 5.2)		7.8 (± 4.7)	7.7 (± 4.7)
Number of Participants with ≥ 1 persisting Symptom at BV (first Infection)			92 (54%)		502 (46%)	594 (47%)
Number of persisting Symptoms per Participant (first Infection) (mean)			2.6 (± 3.1)		2.2 (± 2.9)	2.3 (± 3.0)
Severity of Impairment (first Infection) (mean)			3.6 (± 3.0)		2.8 (± 2.9)	2.9 (± 2.9)
Number of Participants with ≥ 1 persisting Symptom at BV (all Infections)			136 (80%)		769 (71%)	905 (72%)
Number of persisting Symptoms per Participant (all Infections) (mean)			3.2 (3.7)		2.6 (3.4)	2.7 (3.4)
Severity of Impairment (all Infections) (mean)			3.9 (± 3.0)		2.8 (2.8)	2.9 (± 2.9)
Interval 1st Infection – BV (median days; Q1-Q3)			385 (287–497)		381 (322–441)	382 (317–445)
*Number of Participants per Study Center*						
Center 1			10 (6%)		245 (23%)	255 (20%)
Center 2			16 (9%)		173 (16%)	189 (15%)
Center 3			52 (31%)		149 (14%)	201 (16%)
Center 4			71 (42%)		96 (9%)	167 (13%)
Center 5			11 (7%)		178 (16%)	189 (15%)
Center 6			9 (5%)		247 (23%)	256 (20%)

*BV* baseline visit

**Fig. 2:**
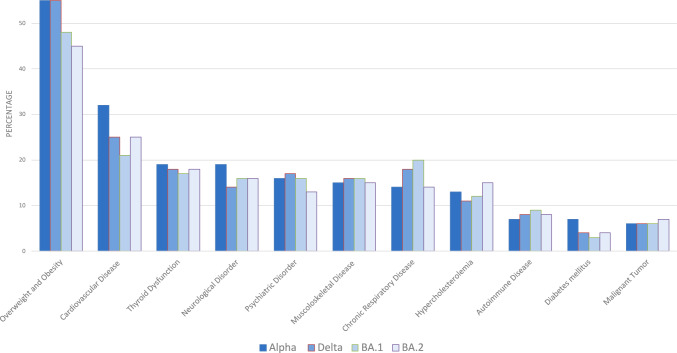
Frequency of pre-existing conditions regarding the VOC

Regarding pre-existing conditions, the most common comorbidity was overweight and obesity (48.9%), followed by cardiovascular diseases (24.9%), thyroid dysfunctions (17.6%), neurological disorders (17.6%), chronic respiratory diseases (15.9%) (Fig. 2). Regarding pre-existing conditions in different VOCs, we excluded BA.5 due to the low number of overall participants and the lack of appropriate statistical subgroup analyses.

### Stratification by SARS-CoV-2 Wave (VOC)

Participants were stratified into five subgroups based on their positive SARS-CoV-2 test date and the corresponding VOC in Germany, Table 3. The highest number of participants was enrolled in group 4 (infection period CW 09/2022 – 21/2022), representing the Omicron BA.2 variant, with a total of 436 (34.6%) participants, while the lowest number of participants were included in the Omicron BA.5 variant (infection period starting with CW 22/2022) with a total of 29 (2.4%) participants, Table 1. Regarding the proportions of different VOCs, study center 6 recruited comparatively fewer participants with the Alpha variant (9% (n = 19) of all participants with Alpha variant) and a higher number with the BA.2 variant (31% (n = 137) of all participants with BA.2 variant), whereas study center 4 recruited fewer participants with the BA.2 variant (3% (n = 15) of all participants with BA.2 variant), Table 1.

**Table 3:** Classification to describe the COVID-19 Pandemic events in Germany, 2020–2022; VOC: Variant of Concern; adapted from [32]

Event	Period (calender week/year)
*Phase 1–3:* First Covid-19 Wave (wild-type); Summer Plateau 2020; Second Covid-19 Wave (wild-type);	10/2020–8/2021
*Phase 4–5:* Third Covid-19 Wave (VOC: Alpha); Summer-Plateau 2021;	9/2021–30/2021
*Phase 6:* Fourth Covid-19 Wave (VOC: Delta)	31/2021–51/2021
*Phase 7: Fifth Covid-19 Wave*	
VOC: Omicron BA.1	52/2021–8/2022
VOC: Omicron BA.2	9/2022–21/2022
*Phase 8:* Sixth Covid-19 Wave (VOC: Omicron BA.5)	22/2022–5/2023

### Ongoing symptoms and severity of impairment

During infection, the mean number of symptoms was 7.7 (± 4.7), while at the time of BV an overall mean number of symptoms of 2.7 (± 3.4) was reported, Table 1. The mean number of persisting symptoms per participant was the lowest in participants with BA.2 (1.8 ± 2.6), followed by BA.2 (2.3 ± 2.7). The percentage of participants with at least one persisting symptom at the BV were higher for participants with Alpha, Delta and BA.5, compared to the participants with BA.1 and BA.2. This was the case regarding the first infection (Alpha: 51% (n = 111), Delta: 51% (n = 170), BA.5: 66% (n = 19)) compared to the participants with BA.1 (45%, n = 108) and BA.2 (43%, n = 186) and regarding all infections (Alpha: 87% (n = 190), Delta: 82% (n = 274), BA.5: 76% (n = 22); on the other hand BA.1 (68%, n = 163) and BA.2 (59%, n = 256), Table 1. While the mean number of persisting symptoms at baseline showed no strong differences between waves of infection, the severity of impairment due to persisting symptoms was the highest in participants having their first infection in the early pandemic, Table 1.

### Frequency of persistent symptoms regarding baseline parameter, the number of infections, and VOC by Multivariable Regression

Persistent symptoms at baseline were analysed by multivariable regression models, Table 4. Female sex (OR: 1.50; 95% CI: 1.14–1.97, p = 0.004), low education (OR: 2.30; 95% CI: 1.71–3.10, p =  < 0.001), and a shorter period between infection and baseline examination in months (OR: 0.92, 95% CI: 0.88–0.96, p =  < 0.001) were associated with the odds of reporting persisting symptoms at baseline. Odds of reporting persistent symptoms also differed by study center. Vaccination status and the number of previous infections did not seem to affect the presence of persistent symptoms. There were differences regarding the VOCs and the chance of reporting persistent symptoms: Omicron BA.1 (OR: 0.28, 95% CI: 0.12–0.7, p = 0.01) and Omicron BA.2 (OR: 0.21, 95% CI: 0.08–0.5, p =  < 0.01) revealed a much lower odds for persisting symptoms than SARS-CoV-2 Alpha variant, Table 4.

**Table 4:** Probability of persisting symptoms regarding baseline parameter, study center, the number of infections, and wave (multivariable logistic regression; response variable: persistent symptoms yes/no)

Variable	OR (95% CI)	p-value
Age (years)	1.01 [0.99;1.02]	0.27
Sex (female vs. male)	1.5 [1.14;1.97]	0.004
Low education	2.3 [1.71;3.1]	< 0.001
4th Wave vs. 1st–3rd Wave VOC: Delta vs. Alpha	0.67 [0.28;1.6]	0.36
5.1th Wave vs. 1st–3rd Wave VOC: Omicron BA.1 vs. Alpha	0.28 [0.12;0.7]	0.01
5.2th Wave vs. 1st–3rd Wave VOC: Omicron BA.2 vs. Alpha	0.21 [0.08;0.5]	< 0.001
6th Wave vs. 1st–3rd Wave VOC: Omicron BA.5 vs. Alpha	0.36 [0.1;1.31]	0.12
≥ 2 Infections	1.04 [0.75;1.45]	0.8
Vaccination before 1st Infection yes vs. no	0.91 [0.38;2.17]	0.83
Center 2 vs. Center 1	0.69 [0.43;1.09]	0.11
Center 3 vs. Center 1	0.8 [0.49;1.31]	0.38
Center 4 vs. Center 1	0.99 [0.58;1.68]	0.96
Center 5 vs. Center 1	1.62 [0.99;2.67]	0.06
Center 6 vs. Center 1	0.77 [0.5;1.19]	0.24
Interval 1st Infection to BV (month)	0.92 [0.88;0.96]	< 0.001

*OR* odds ratio,* VOC* variant of concern,* CI* confidence interval, * Center 1* University Hospital of Düsseldorf, * Center 2* University Hospital of Aachen, * Center 3* University Hospital of Bonn, * Center 4* University Hospital of Essen, * Center 5* University Hospital of Cologne, * Center 6* University Hospital of Münster,* BV* Baseline Visite

### Severity of ongoing symptoms regarding baseline parameter, the number of infections, and VOC by multivariable regression

The severity of persistent symptoms was analysed via multivariable linear regression models. Age (β: 0.009, 95% CI – 0.003 to 0.02, p = 0.14), the number of infections (one vs. ≥ 2 infections (β: – 0.128, 95% CI – 0.526 to – 0.27, p = 0.53), and vaccination before the first infection (β: – 0.187, 95% CI – 1.038 to – 0.664, p = 0.64) did not show indication for an association with the severity of persistent symptoms. A longer time period between infection and baseline examination was weakly associated with lower severity of persistent symptoms (β – 0.06 per month, 95% CI – 0.11 to 0.01, p = 0.02). Instead, lower education level (β: 1.184, 95% CI: 0.854–1.515, p =  < 0.001) and female sex (β: 0.567, 95% CI: 0.25–0.883, p =  < 0.001) were associated with greater severity of persistent symptoms. Regarding the VOC, all Omicron variants (BA.1: β – 1.276, 95% CI – 2.169 to 0.384, p =  < 0.01; BA.2: β: – 1.518; 95% CI – 2.387 to – 0.649, p =  < 0.001; BA.5: β: – 1.36; 95% CI – 2.704 to – 0.015, p = 0.048) showed lower severity of persistent symptoms compared to the Alpha variant. Participants of the Delta wave tended to have severity of persistent symptoms in between the Alpha and Omicron variants, but the association was less strong (Delta vs. Alpha: β: – 0.536, 95% CI – 1.395 to – 0.323, p = 0.22), Table 5.

**Table 5:** The severity of persistent symptoms (multivariable linear regression; response variable: severity of ongoing symptoms, on a scale 0–10)

Variable		β (95% CI)		p-value
Age (years)		0.009 [– 0.003;0.02]		0.14
Sex (female vs. male)		0.567 [0.25;0.883]		< 0.001
Low education		1.184 [0.854;1.515]		< 0.001
4th Wave vs. 1st–3rd Wave VOC: Delta vs. Alpha		– 0.536 [– 1.395; – 0.323]		0.22
5.1th Wave vs. 1st–3rd Wave VOC: Omicron BA.1 vs. Alpha		– 1.276 [– 2.169; – 0.384]		0.005
5.2th Wave vs. 1st–3rd Wave VOC: Omicron BA.2 vs. Alpha		– 1.518 [– 2.387; – 0.649]		< 0.001
6th Wave vs. 1st–3rd Wave VOC: Omicron BA.5 vs. Alpha		– 1.36 [– 2.704; – 0.015]		0.048
≥ 2 Infections		– 0.128 [– 0.526;0.27]		0.53
Vaccination before 1st Infection yes vs. no		– 0.187 [– 1.038;0.664]		0.67
Center 2 vs. Center 1		– 0.371 [– 0.914;0.172]		0.18
Center 3 vs. Center 1		– 0.409 [– 0.966;0.15]		0.15
Center 4 vs. Center 1		0.13 [– 0.466;0.727]		0.67
Center 5 vs. Center 1		0.822 [0.294;1.351]		0.002
Center 6 vs. Center 1		– 0.538 [– 1.039; – 0.037]		0.04
Center 2 vs. Center 1		– 0.371 [– 0.914;0.172]		0.18
Interval 1st Infection to BV (month)		– 0.06 [– 0.11; – 0.01]		0.02

*β* beta estimate,* VOC* variant of concern,* CI* confidence interval, * Center 1* University Hospital of Düsseldorf, * Center 2* University Hospital of Aachen, * Center 3* University Hospital of Bonn, * Center 4* University Hospital of Essen, * Center 5* University Hospital of Cologne, * Center 6* University Hospital of Münster,* BV* Baseline Visite

### Frequency of different persisting symptoms regarding different VOCs

**Fig. 3:**
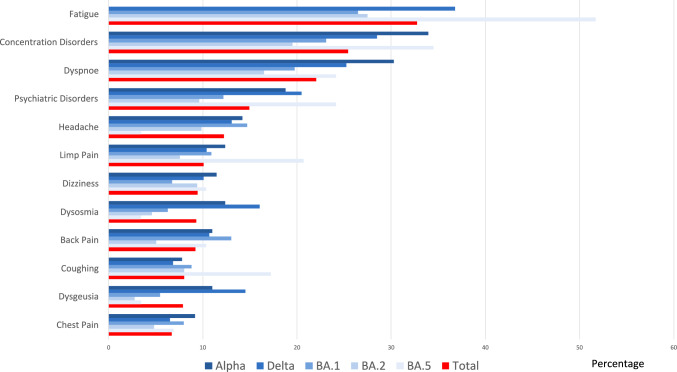
Frequency of ongoing symptoms regarding different VOCs

**Fig. 4:**
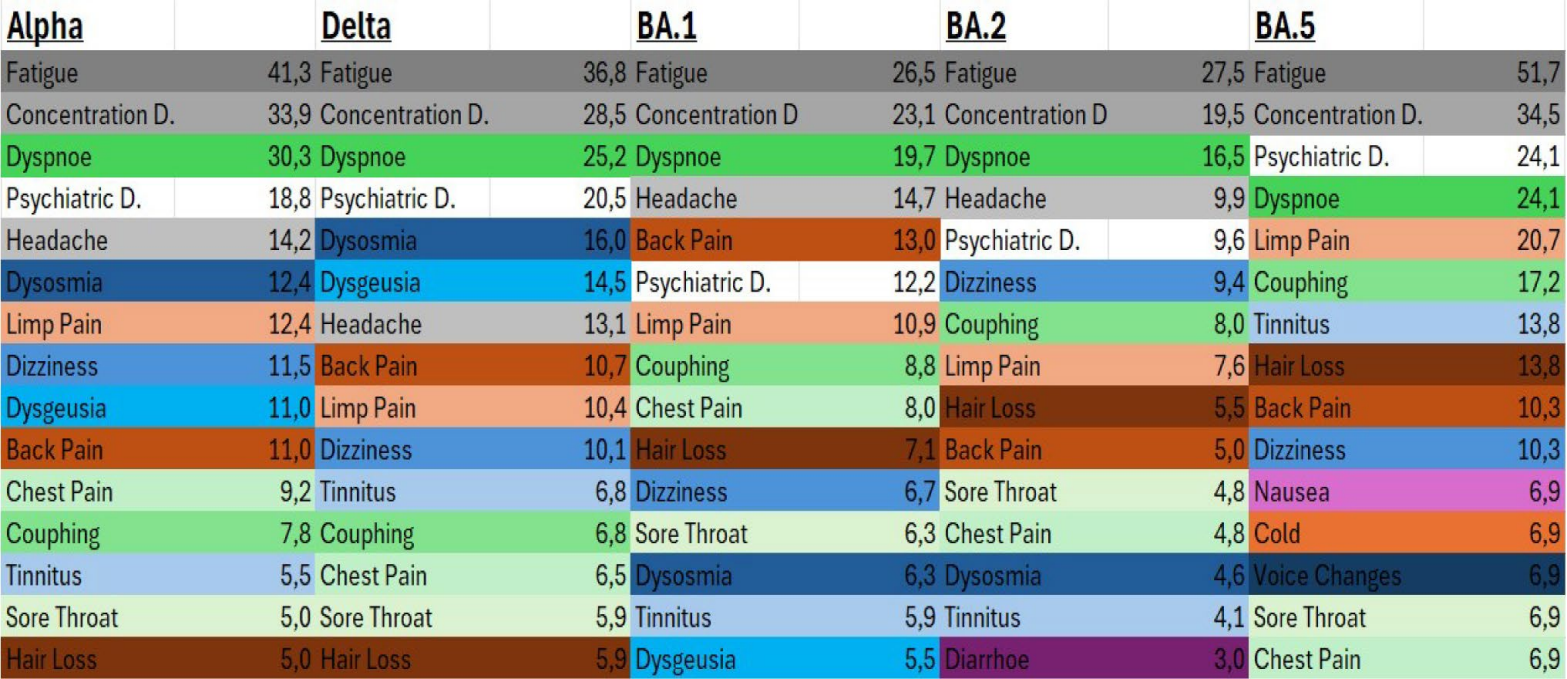
Frequency of ongoing symptoms regarding the VOC and symptom clusters, showing the 15 most frequent symptoms per VOC. grey shades: chronic fatigue-like (fatigue, headache, concentration disorders); green shades: respiratory (dyspnoea, cough, chest pain, sore throat, voice change); blue shades: neurosensorial (dysgeusia, dysosmia, dizziness, tinnitus); purple shades: gastrointestinal (nausea, diarrhoe); brown shades: systemic-inflammatory chronic pain (limb pain, back pain, cold; hair loss)

Fatigue was the most common symptom throughout all virus variants (Alpha, Delta, Omicron BA.1, BA.2, and BA.5) with an overall frequency of 32.8% of all participants at BV (Fig. 3). Concentration disorders (25.4%) and dyspnoea (22%) followed as most common persisting symptoms in all VOC (Fig. 3, Fig. 4). Fatigue was less frequently reported in Omicron BA.1 (26.5%) and BA.2 (27.5%) compared to the Alpha (41.3%) and Delta (36.8%) variants (Fig. 2, Fig. 3). Concentration disorders were less frequent in Omicron BA.1 (23.1%) and BA.2 (19.5%) compared to Alpha (33.9%) and Delta (28.5%). Dyspnoea was also less common in BA.1 (19.7%) and BA.2 (16.5%) compared to Alpha (30.3%) and Delta (25.2%). Dysgeusia and Dysosmia were both less common in BA.1 and BA.2. compared to Alpha and Delta. Psychiatric disorders were less frequent in BA.1 (12.6%) and BA.2. (9.6%) compared to Alpha (18.8%) and Delta (20.5%) variants (Figs. 3, 4). Regarding total differences between the VOC, Alpha and Delta variants showed generally similar frequencies of persisting symptoms.

### Frequency of persisting symptoms regarding VOC and symptom-cluster

**Fig. 5:**
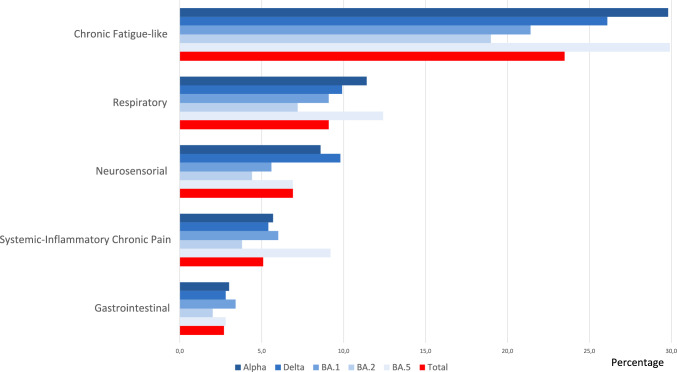
Frequency of ongoing symptoms regarding different VOCs and symptom clusters as a percentage of each cluster and corresponding VOC

Regarding different symptom cluster, chronic fatigue-like syndrome was with 23.5% the most frequent cluster, followed by respiratory cluster (9.1%), neurosensorial syndrome cluster (6.9%), systemic-inflammatory chronic pain syndrome cluster (5.1%), and gastrointestinal cluster (2.7%) (Figs. 4, 5). Regarding different VOCs, chronic fatigue-like cluster was more frequent in Alpha (29.8%) and Delta (26.1%) variants compared to Omicron BA.1 (21.4%) and BA.2 (19%) (Fig. 5). The respiratory cluster was more common in Alpha (11.4%) compared to Omicron BA.1 (9.1%) and BA.2 (7.3%). Delta (9.9%) had respiratory cluster frequency between Alpha and BA.1/BA.2. The gastrointestinal cluster showed overall (2.7%) low and constant frequencies throughout all VOCs (Alpha: 3%, Delta: 2.8%, BA.1: 3.5%; BA.2: 2.8%; BA.5: 2.8%). The systemic-inflammatory chronic pain cluster was present in 5.1% overall. BA.2 (3.8%) showed a lower frequency compared to Alpha (5.7%), Delta (5.4%), and BA.1 (6%). BA.5 had the highest percentage of participants with systemic-inflammatory chronic pain cluster (9.2%) (Fig. 5).

## Discussion

Data analyses revealed considerable differences in reporting persisting symptoms as well as subjective symptom severity at study baseline regarding different VOCs. In general, post COVID symptoms became less frequent with each new variant of concern. It is essential to mention that comparing epidemiological studies on post COVID is challenging, as they have widely different recruitment strategies, follow-up periods, and heterogenous participant demographics [33–39]. This is particularly the case regarding frequency, timeline, and severity of symptoms, especially given the natural course of post COVID per se, with fluctuating and relapsing symptoms and symptom severity over time [40]. In our study, 72% of participants reported at least one persisting symptom at BV individually attributed to SARS-CoV-2, with a higher proportion of symptomatic participants in the hospitalized sample than the population sample (80% vs. 71%). One year after infection, the reported rates of symptomatic patients are extensively heterogeneous, with a 30–60% range in most published results [12, 39, 41–45]. According to our data, female sex, lower education, and a shorter period between infection and BV were associated with higher rates of persisting symptoms and were independent predictors of symptom severity in multivariable regression models. This is in line with previously published data [46]. Interestingly, we report no age effect either on the number of symptoms or regarding symptom severity. While most of the published post COVID studies reported higher risk in elderly patients, several studies found no age effect or only an effect in specific age subgroups (e.g., > 50 years) [47, 48]. We have to state that we did not include participants > 75 years, and therefore our results are not transferable for this age group. Furthermore, most of the studies did not record the severity of symptoms. Regarding the influence of age on symptom frequency, a recent review stated a high heterogeneity rate especially in large studies, and that high-quality studies have higher heterogeneity [46]. Along with our results, age per se is no risk factor for post COVID. In multivariable regression, vaccination status was not an independent risk factor for persisting symptoms and symptom severity. The vast majority (84%) in our study were vaccinated before the first infection; therefore, the statistical power to detect a possible protective effect is very low. This can be ascribed to the recruitment phase (infections after March 1st, 2021) and the high vaccination coverage in Germany, with an exponential increase in vaccinations between March and July 2021 [49]. Furthermore, persons refusing SARS-CoV-2 vaccination are disproportionately often skeptical about COVID-19 effects and/or healthcare authorities and, therefore, may be less interested in participating in a study trial [50–52].

Hospitalized participants tended to have more persistant symptoms at BV compared to participants of the population sample and had a higher severity of impairment. Several studies, including a meta-analysis, showed a higher prevalence of post COVID in hospitalized patients than in outpatients [16, 53, 54]. Regarding different virus variants, various studies, including meta-analyses, have investigated the risk of post COVID with inconclusive results [13, 19, 24, 55, 56]. The available data suggest that infection with an Omicron variant may result in fewer post COVID symptoms. However, since most studies compared only two different virus variants and heterogeneous patient collectives, transferability on all variants is difficult [18, 57, 58]. Fernandes-de-la-Pena et al. concluded in their review that the small number of studies and the lack of control of confounders, e.g., reinfections or vaccine status, limit the generalizability of the results [55]. Vishwakarma et al. stated that the difference in the prevalence of post COVID between various variants needs further exploration [13]. Pathophysiological differences, e.g., higher viral load (Delta > Alpha), higher transmissibility (Omicron > Alpha and Delta), and potential immune escape to vaccines among the different VOCs, were reported [59–61]. Our findings suggest that Alpha and Delta, on the one hand, and Omicron BA.1 and BA.2, on the other hand, have highly homogenous frequencies and distribution of persisting symptoms (single-symptoms as well as symptom-clusters). We observed a lower rate of persistent symptoms in the Omicron variants BA.1 and BA. 2 compared to the Alpha and Delta variants. Besides possible differences in the viral variants themselves, this may also be explained by the increasing immunity of the population against SARS-CoV-2, which is a result of a higher number of vaccinations and natural infections in the course of the pandemic. Omicron BA.5 participants tended to have a higher symptom probability than all other VOCs, but the results were overall not significant. This may be attributable to the low number of participants in BA.5. We assume that the shorter period between infection and BV is the main reason for this finding. Subsequent analyses of our cohort regarding long-term follow-up will help clarify this.

Overall (all VOCs), fatigue was the most frequent ongoing symptom (32.8%), followed by concentration disorders (25.4%) and dyspnoea (22%). This is in line with previously published data, including a systematic review reporting rates of 41% for fatigue and 31% for dyspnoea 12 months after infection but making no distinction between different VOCs [12]. Fatigue, concentration disorders, and the chronic fatigue-like symptom cluster, as well as dyspnoea, were significantly less frequent in Omicron BA.1 and BA.2 compared to the Alpha and Delta variants. This finding contrasts with previously published data [19, 27, 55]. A series of symptoms (headache, dizziness, tinnitus, sore throat, nausea, loss of appetite, diarrhoea, abdominal pain, and hair loss) showed consistent frequencies throughout all VOCs in our data. After Omicron infection (BA.1, BA.2, and BA.5), participants were less affected by smell and taste disorders compared to Alpha and Delta, which confirms recently published data from a digital participation-only study based on self-reports [62]. Our data also show a lower frequency of the whole neurosensorial symptom cluster (dysgeusia, dysosmia, dizziness, tinnitus, earache) in BA.1 and BA.2 compared to Alpha and Delta variants. However, it must be pointed out that we did not categorize psychiatric disorders into a specific cluster due to the large heterogeneity in this generic term. Furthermore, we have to state that we did not perform cluster analyses based on our own data and therefore symptom allocation to previously described clusters in our study sample remains debatable. While men are at higher risk for severe COVID-19 and death, women appear more prone to developing post COVID [38, 63, 64], consistent with most published studies [65, 66]. Sex specific difference in the response to infections are described in many contexts. Recent studies showed sex-specific immune responses, with females exhibiting more severe inflammation possibly attributing to differences in regard to the likelihood and phenotype of post COVID [67–69]. The higher proportion of female participants in our study may also reflect women’s generally higher response and participation rates in clinical research [70]. 32% of participants in our collective had more than one infection at BV, slightly higher than the reported rates in population-based analyses with 5–15% [71, 72]. The interval between infection and BV in the study centers in our study was overall roughly a year with a median of 382 (317–445) days. Therefore, the BV represents a comparatively late time point after the initial SARS-CoV-2 infection in most participants.

This study has several limitations. Despite random symptom-unrelated selection, it is probable that symptomatic patients followed the study invitation more frequently, and therefore, bias may be present with a possible overestimation of frequency and severity of symptoms by an overrepresentation of symptomatic individuals. It is also evident that the response rate was rather low, questioning the generalisability of findings. Furthermore, some reported symptoms are subjective (e.g., fatigue, headache), and the lack of validated scales to measure most symptoms makes it difficult to compare data between subjects. Furthermore, we could not discriminate between different virus variants per PCR test. Thus, the allocation to a specific VOC in our cohort is based on the infection time.

In summary, our results add relevant data regarding symptoms and symptom severity caused by the different virus variants in hospitalized and non-hospitalized participants stratified by different VOCs, demographic, and educational participant-related context factors. Further follow-up analyses of our cohort, with its three-year horizon, including laboratory tests and standardized medical examinations, will help characterize the risk factors and natural course of post COVID. Furthermore, planned collaborations (e.g., with the German National Cohort (NAKO), which will generate a control group for our study sample from the general population) will strengthen and expand the relevance of our data.

## Data Availability

The results of the remote data analyses are available from the corresponding author upon reasonable request. Contact: Dr. med. Alexander Killer; E-Mail: alexander.killer@med.uni-duesseldorf.de.

## Declarations

### Study centres

The coordinator center is the Department of Gastroenterology, Hepatology and Infectious Diseases, University Hospital, Medical Faculty of Heinrich Heine University Düsseldorf, Germany. Further centers are the Department of Pneumology and Intensive Care Medicine, RWTH University Hospital, Aachen, Germany; Department of Medicine I, University Hospital Bonn, Germany; Department of Infectious Diseases; West German Centre of Infectious Diseases, University Hospital Essen, University of Duisburg-Essen, Essen, Germany; Department I of Internal Medicine, Faculty of Medicine and University Hospital, University of Cologne, Germany; Department of Internal Medicine B, Gastroenterology, Hepatology, Endocrinology and Clinical Infectiology, University Hospital Münster, Germany.

### Clinical trial registration

This study was registered in the German Clinical Trials Register under the Clinical trial number DRKS00027377 on 08.12.2021, accessible online at: https://drks.de/search/en/trial/DRKS00027377/details

### Data collection

Pseudonymized health data was collected by the study centers during each study visit on standardized case report forms (CRFs). This included demographic, clinical, and viro-immunological data. These health data were transferred into a clinical database system (Clincase, Quadratek Data Solutions Ltd., Berlin, Germany), administered by the Center for Clinical Studies Essen, Germany (ZKSE). Recording and health data transfer were subject to the consent of the study participants. Data collection procedures strictly adhere to the established protocol, the recent version of the Declaration of Helsinki, general data protection regulations of the European Union (EU-DSGVO), and the International Conference on Harmonization Good Clinical Practice (ICH-GCP). Data quality and plausibility checks were carried out by trained ZKSE employees. Complex plausibility checks were programmed in SAS. If necessary, queries were sent to members of the study teams at the centers to make corrections to the data.

### Data protection

The pseudonymously collected data is analyzed and evaluated for interim evaluations and finally for the legally required evaluations. All data in the study database can only be identified by a participant identification number (participant ID) corresponding to participant consent to ensure pseudonymization. The birth year is documented in the database, not the day and month. Only the local study centers maintain a separate confidential identification list to identify everyone enrolled to enable participants. This list is protected from unauthorized access stored in the respective study center. All persons who have access to pseudonymized data are subject to the general data protection regulations of the European Union (EU-DSGVO) when handling the data. Only anonymized data are passed on when the final study report is submitted to the responsible German authorities (higher federal authorities, ethics committees if applicable). Publications on the study will only be made with anonymous data. The data must be retained following legal requirements. An assessment of the necessity and proportionality of the data processing was obtained. A risk assessment regarding the possible risks caused by the processing for those affected is taken into account, and the protection goals of confidentiality, integrity, and availability were assessed. The remaining residual risk regarding the protection goals of integrity, legal control, availability, and confidentiality after implementation of technical and organizational measures are classified as low.
